# Rethinking Resting Heart Rate Variability: No Evidence of Association With Self‐Regulation and Psychopathology in a Cross‐Sectional Study Among Adolescents in Colombia, Nepal, and South Africa

**DOI:** 10.1111/psyp.70184

**Published:** 2025-11-14

**Authors:** Amin Sinichi, Georgia Eleftheriou, Mai Anh Ha Ngoc, Brandon A. Kohrt, Nagendra P. Luitel, Rakesh Singh, Roxanne Jacobs, Katherine Sorsdahl, Sandra Garcia Jaramillo, Maria Cecilia Dedios Sanguineti, Crick Lund, Mark Jordans, Emily Garman, Lydia Krabbendam, Martin Gevonden

**Affiliations:** ^1^ Department of Clinical, Neuro‐ & Developmental Psychology, Faculty of Behavioural and Movement Sciences Vrije Universiteit Amsterdam Amsterdam the Netherlands; ^2^ Institute Brain and Behaviour (iBBA) Amsterdam the Netherlands; ^3^ Center for Global Mental Health Equity, Department of Psychiatry and Behavioral Health George Washington University Washington DC USA; ^4^ Research Department Transcultural Psychosocial Organization Nepal (TPO Nepal) Kathmandu Nepal; ^5^ Centre for Global Mental Health, Health Service and Population Research Department Institute of Psychiatry, Psychology and Neuroscience, King's College London London UK; ^6^ Alan J. Flisher Centre for Public Mental Health, Department of Psychiatry & Mental Health University of Cape Town Rondebosch South Africa; ^7^ School of Government Universidad de los Andes Bogotá Colombia; ^8^ Department of Mental Health Johns Hopkins Bloomberg School of Public Health Baltimore Maryland USA; ^9^ Department of Biological Psychology, Faculty of Behavioural and Movement Sciences Vrije Universiteit Amsterdam Amsterdam the Netherlands

## Abstract

Resting‐state heart rate variability (HRV) has been proposed by some researchers as a potential transdiagnostic measure of psychopathology. It is often found to be reduced across a range of mental health conditions and is thought to reflect self‐regulatory processes, which are often impaired in these conditions. However, most existing evidence is derived from high‐income countries with limited ethnic variation. This leaves a critical gap in understanding whether these associations generalize to low‐ and middle‐income countries (LMICs) or to individuals from under‐represented ethnic backgrounds. In this cross‐sectional study, we examined the relationship between resting‐state HRV, self‐regulation, and psychopathology in a large, diverse sample of 1107 adolescents aged 13–15 years living in urban poverty across Colombia, Nepal, and South Africa. We tested seven pre‐registered hypotheses across two domains: self‐regulation (emotional regulation, inhibitory control, and delay discounting) and psychopathology (anxiety, depression, externalizing symptoms). We used linear mixed models for the confirmatory analyses and complemented them with exploratory equivalence testing. Contrary to our hypotheses based on the neurovisceral integration (NVI) model, HRV was not significantly associated with any outcomes, except for a small effect on externalizing behavior in the opposite direction of our hypothesis. Equivalence testing further indicated that the observed estimates were statistically equivalent, which suggests a meaningful effect is absent. These findings challenge the assumption that HRV–psychological associations are universal and point to the importance of considering contextual, cultural, developmental, and methodological factors in psychophysiological research. This study aims to contribute to a broader rethinking of HRV's role in mental health research, especially in populations that have been under‐represented in the literature.

## Impact Statement

This study contributes to a broader rethinking of heart rate variability as a psychophysiological index by examining its associations with self‐regulation and psychopathology in adolescents living in poverty in Colombia, Nepal, and South Africa. The absence of meaningful relationships between HRV and psychological outcomes highlights the importance of considering cultural, contextual, developmental, and methodological factors in HRV research and cautions against universal assumptions drawn from high‐income country samples.

## 1 Introduction

### 1.1 Background

Adolescence is a critical developmental period when the nervous system, particularly brain networks involved in self‐regulatory mechanisms, is still maturing (Best and Miller 2010; Blakemore and Choudhury 2006; Hackman and Farah 2009). It is also marked as the peak period for the onset of most psychopathologies (Paus et al. 2008; Solmi et al. 2022). Globally, the majority (90%) of the world's adolescent population resides in low‐ and middle‐income countries (LMICs), where one‐third live in multidimensional poverty: households deprived of financial resources, education, or access to essential infrastructure and services (Alkire et al. 2019). Growing up and living in such socioeconomic adversity is a significant risk factor for developing psychopathology, such as depression and anxiety (Lorant et al. 2003; Lund et al. 2010, 2011; Reiss 2013).

Although adolescence is a sensitive period for psychopathology and socioeconomic adversity is a well‐known risk factor, the mechanisms linking the two remain poorly understood. Among several proposed pathways, one potential mechanism is the impact of socioeconomic adversity on the development and functioning of the autonomic nervous system (ANS). Greater socioeconomic adversity across the life course is shown to be associated with higher ANS allostatic load in adulthood (Gruenewald et al. 2012). Similarly, childhood adversity more broadly has been linked to blunted stress‐related autonomic reactivity later in life (Voellmin et al. 2015) and lower baseline vagal activity, specifically in those diagnosed with a psychiatric disorder (Wesarg et al. 2022). Other studies have also shown that low socioeconomic status (SES) is associated with less adaptive trajectories of ANS functioning, such as consistently higher heart rates and greater sympathetic activation in young children (Johnson et al. 2017). Busso et al. (2017) further illustrate how adversity in adolescence may contribute to psychopathology through reduced physiological reactivity, which could foster sensation seeking and related behaviors, which are specifically relevant in the development of externalizing disorders (Loheide‐Niesmann et al. 2021).

One noninvasive way to investigate altered autonomic functioning is through heart rate variability (HRV), which quantifies the variation in cardiac beat‐to‐beat intervals (heart periods, or HP). At rest, this variability in heart periods is primarily regulated via the vagus nerve, whose tonic firing pattern is modulated by the phases of respiration; a phenomenon known as respiratory sinus arrhythmia (RSA) (Pomeranz et al. 1985; Grossman and Taylor 2007; Katona and Jih 1975), or more recently renamed respiratory heart rate variability, or RespHRV (Menuet et al. 2025). RSA/RespHRV can be approximated by some HRV metrics, such as high‐frequency HRV (HF‐HRV) and the root mean square of successive differences (RMSSD) (Berntson et al. 2005; Lewis et al. 2012; Penttilä et al. 2001; Quigley et al. 2024; Shaffer and Ginsberg 2017). HRV can be measured with noninvasive technologies at low cost, which is especially valuable when doing research in resource‐limited LMIC contexts, such as for investigating the potential role of poverty‐induced autonomic dysregulation in the development of psychopathology.

Given that HRV offers a noninvasive proxy for cardiac parasympathetic control, it has become central to several theoretical frameworks that link autonomic function to psychological processes and/or the development of psychopathology (Grossman and Taylor 2007; Thayer et al. 2009; Porges 2007; Lehrer and Gevirtz 2014; McCraty and Childre 2010; Laborde et al. 2018). Among them, the neurovisceral integration (NVI) model specifically focuses on how HRV, self‐regulatory mechanisms, and psychopathology relate to one another (Thayer et al. 2009; Thayer and Lane 2000, 2009), with an emphasis on the role of cortical control over cardiac activity. According to NVI, a shared neural network known as the central autonomic network (CAN; with a prominent role for the prefrontal cortex, PFC) exerts top‐down inhibitory control over cognitive, emotional, and physiological functions, with HRV serving as an “index” of this network. In other words, since the same neural structures are presumed to regulate these domains, the model hypothesizes that higher resting‐state HRV is associated with stronger self‐regulatory mechanisms, as HRV reflects the PFC's inhibitory control. On the basis of this PFC–HRV link, the model further proposes that, given the PFC's involvement in most psychopathologies, HRV can serve as a transdiagnostic measure of psychopathology (Beauchaine and Thayer 2015), such that lower resting‐state HRV may be generally linked to a greater risk of developing mental health conditions. The model specifically emphasizes resting‐state HRV as a trait‐like variable associated with individual differences in cognitive functioning and physical and mental health outcomes, and also because of the relative stability of resting‐state HRV over time (Thayer et al. 2009).

Previous research, predominantly conducted in high‐income countries (HICs), provides initial evidence supporting the NVI model. Meta‐analyses and systematic reviews report associations between HRV and various PFC‐dependent cognitive functions (though the reported effect sizes tend to be small), such as global cognition, memory, language, attention, executive function, and processing speed (Forte et al. 2019; Nicolini et al. 2024), as well as top‐down self‐regulation (Holzman and Bridgett 2017) and self‐control (Zahn et al. 2016). Furthermore, meta‐analyses have established an association between HRV and psychopathology, including psychiatric disorders (Ramesh et al. 2023) such as anxiety (Chalmers et al. 2014; Cheng et al. 2022), depression in adults (Brown et al. 2018) and in children and adolescents (Ding et al. 2024), and stress (Kim et al. 2018), to name only a few. Taken together, the NVI model proposes HRV as an index of top‐down self‐regulatory mechanisms. Given the role of impaired self‐regulation in the development of psychopathologies, particularly as a consequence of adversities such as poverty (Lund et al. 2023; Merz et al. 2019; Palacios‐Barrios and Hanson 2019; Wesarg et al. 2020), HRV has gained interest as a potential independent variable, or even as a target (a dependent variable), for intervention through methods such as biofeedback or noninvasive vagus nerve stimulation. These applications rely on the assumption of the NVI model that the associations between HRV, psychopathology, and self‐regulation are universal, and that HRV is consistently predictive across diverse contexts and populations.

Subsequent studies have challenged this universality assumption by identifying context and population‐specific aspects of the relationship between HRV, self‐regulation and psychopathology (Jennings et al. 2015; McGinley et al. 2018; Watanabe, Tyra, et al. 2025). Factors such as age, ethnicity, and SES appear to moderate these associations. For instance, developmental trajectories of cortical and autonomic functions may underlie stronger HRV–self‐regulation links in older individuals compared to younger ones (Holzman and Bridgett 2017). Ethnicity also plays a role; Jennings et al. (2015) reported differential associations between HRV and cortical blood flow among Caucasian and African American participants. SES further complicates these relationships, as shown in findings where higher HRV correlates with the ability to delay gratification in high‐SES children but shows an inverse relationship in low‐SES groups (Sturge‐Apple et al. 2016). Such evidence aligns with evolutionary‐developmental models suggesting that the behaviors that may be considered adaptive vary with environmental stability and resource availability (Daly and Wilson 2005; Ellis et al. 2009).

Taken together, there is emerging evidence suggesting a more context‐ or population‐dependent relationship, rather than a universal link, between HRV, self‐regulation, and psychopathology. This interplay appears to be nuanced and potentially moderated by environmental and sociodemographic factors which are not yet fully understood. The current study is the first to investigate these relationships at scale in LMIC settings facing high socioeconomic adversity, which allows us to further test the assumption of universality proposed by the NVI model. Regardless of the outcome regarding that assumption, it will improve our understanding of the psychophysiology of the global adolescent population.

### 1.2 Current Study

The current study explores the relationship between HRV, self‐regulation, and psychopathology among adolescents living in urban poverty in Colombia (Bogotá), Nepal (Kathmandu), and South Africa (Cape Town). The sample is drawn from the cross‐sectional data collection phase of the ALIVE research project (Lund et al. 2023). This study focuses on pooled data from the three countries, and analyzes a subset of selected instruments relevant to the research aims. We analyzed data from 1107 adolescents aged 13–15 years (417 from Colombia, 372 from Nepal, and 318 from South Africa), all recruited through schools located in disadvantaged communities.

In a pre‐registered analysis plan, we assessed two main outcome domains, self‐regulation and psychopathology, in relation to resting‐state HRV (quantified as RMSSD). For the self‐regulation domain, measures included the following: the Difficulties in Emotion Regulation Scale‐Short Form (DERS, Kaufman et al. 2016); the Emotional Go/No‐Go Task (EGNG, Tottenham et al. 2011), with the key variable being false alarms, which have been shown to be specifically elevated in adolescents with depression and anxiety and are indicative of difficulties in inhibitory control (Ladouceur et al. 2006; Schulz et al. 2007); and the Delay Discounting Task (DDT, Richards et al. 1999) which evaluates reward‐based decision‐making, with adolescents with depressive symptoms showing a greater tendency to favor immediate rewards over delayed ones (Felton et al. 2022; Pulcu et al. 2014), consistent with broader evidence identifying steeper delay discounting as a transdiagnostic marker across multiple psychiatric disorders in adults (Amlung et al. 2019). Psychopathology symptoms were assessed using the Measurement of Mental Health among Adolescents and Young People at the Population Level (MMAPP; Carvajal‐Velez et al. 2023), which includes equivalent subscales for anxiety (GAD‐7; Spitzer et al. 2006) and depression (PHQ‐A; Johnson et al. 2002); and finally, the Disruptive Behavior International Scale (DBIS; Burkey et al. 2018) for assessment of externalizing behavior problems.

We hypothesized that, for the self‐regulation domain, lower resting‐state HRV would be associated with: (1) higher emotional dysregulation (DERS scores), (2) more false alarms (poorer inhibitory control) in the EGNG task, and (3) a greater preference for immediate rewards (higher impulsivity) in the DDT. For the psychopathology domain, we expected that lower resting‐state HRV would be associated with: (4) higher anxiety symptoms (GAD‐7 scores), (5) higher depression symptoms (PHQ‐A scores), (6) greater overall psychopathology (total MMAPP scores), and (7) more externalizing behavior problems (DBIS scores). We tested these hypotheses using linear mixed models, adjusting for age, gender, BMI, and Device Type (ECG/PPG), and treated country as a random intercept. Additionally, as an exploratory analysis, we conducted equivalence testing to evaluate the practical significance of observed effect sizes. This study extends prior psychophysiological research by examining whether the NVI model may be generalized to socioeconomically disadvantaged adolescent populations.

## 2 Method

### 2.1 Participants

A total of 1344 adolescents had heart period time series data available, of which 1107 were retained for analysis after preprocessing. These participants (42.36% male, 57.27% female, and 0.36% nonbinary) were part of the cross‐sectional survey of the ALIVE study (Lund et al. 2023). They were recruited from eight public schools in each country, in Bogotá (Colombia), Kathmandu (Nepal), and Cape Town (South Africa), for a total of 24 schools. The adolescents were aged 13–15 years, fluent in the local language (i.e., Spanish, Nepali, and English, respectively), enrolled in public secondary schools, and living in conditions of high poverty based on a priori indicators relevant to each national context (Colombia and South Africa) and a multidimensional poverty screener in Nepal to identify adolescents as living in poor households. Participants were approached in schools, where they received study information and took home assent forms along with consent forms for their caregivers. Their primary caregivers were adults aged 18 or older who consented to participate. The recruitment process was facilitated by local partner organizations: Innovations for Poverty Action (IPA) in Colombia, Transcultural Psychosocial Organization Nepal (TPO Nepal), and the University of Cape Town (UCT) in South Africa.

### 2.2 Ethics

Ethical approval for the study was obtained from King's College London (KCL)'s Health Faculty Research Ethics Subcommittee under reference HR/DP‐23/24‐38680. Approvals were also obtained by each of the data collection sites' Institutional Review Boards: The Faculty of Health Sciences' Human Research Ethics Committee at the University of Cape Town (South Africa) (reference number HREC315/2022), Innovations for Poverty Action's Institutional Review Board (Colombia) (protocol number 4062), and the Ethical Review Board of the Nepal Health Research Council (Nepal) (protocol registration number 661/2023). Participation required both adolescent assent and primary caregiver consent, in compliance with national ethical guidelines in each country. Participants received refreshments during the assessment and a small token (depending on the country, e.g., pen, notebook, grocery voucher) upon completion. Participants experiencing distress had access to trained research assistants who facilitated support and assessed risk levels. Participants identified as having scores above the cutoff based on PHQ‐A or GAD‐7 were provided with a brochure of local mental health services and given access to mental health services upon request. Those with suicidal intent and a plan were immediately referred to clinical staff, such as psychologists, registered counselors, or social workers who conducted further assessments, facilitated urgent referrals, and, if necessary, accompanied them to services.

### 2.3 Procedure

Data collection took place in schools, in a confidential space designated for the study. Data was collected using a Samsung Galaxy Tab A7 (or, in some instances, an A9) Lite. For self‐report data, the ODK platform (https://getodk.org/) was used on the tablets. Neuropsychological tasks were developed as Android applications. Cardiac data was measured using either a Polar H10 chest strap (Polar Electro Oy) or an Inner Balance Coherence Plus earlobe clip (HeartMath Inc.), connected to the HRV Logger Android application (Plews et al. 2017). Research assistants facilitated the completion of self‐reports and neuropsychological tasks and guided participants through the physiological recording procedure. The data collection followed this sequence: first, demographic information such as age and gender was recorded, followed by the administration of the self‐reports. After this period, which also served as the habituation period, resting‐state HRV data were collected, followed by the neuropsychological tasks. Anthropometric (height, weight) measures were obtained either before the administration of self‐reports or after the entire procedure, depending on the country. Additional data was also collected during the sessions, though it falls outside the scope of the current research aims and will not be detailed here (the entire assessment took approximately 90 min to complete). In South Africa, the self‐report instruments were self‐administered in group settings with about 30 participants per group, who were often split into two classrooms, with 4–5 research assistants per classroom. In Colombia and Nepal, the self‐reports were administered individually by the research assistant to each participant, and neuropsychological tasks were self‐administered in all three sites.

### 2.4 Measurement Tools

The instruments were culturally adapted for each site and followed a structured, multi‐stage process as a part of ALIVE adaptation phase (Lund et al. 2023), to ensure linguistic, conceptual, and contextual validity. When required, self‐report tools underwent translation by two independent bilingual translators, followed by expert review by local mental health professionals to assess comprehensibility and cultural appropriateness. Focus group discussions with adolescents (stratified by age and gender) were conducted to refine terminology, clarify ambiguities, and enhance relevance. Back‐translation by independent translators ensured consistency with the original meaning, and cognitive interviews were performed to validate response comprehension and appropriateness. Neuropsychological tasks underwent a similar adaptation process, including expert review of structure, layout, and content, as well as pilot testing among adolescents to assess instruction clarity and task feasibility. Additionally, for the emotional go/no‐go (EGNG) task, which was administered in Colombia and South Africa only, country‐specific photosets of adolescents with the same ethnic background (ages 11–17) expressing different emotions were created, validated in a pilot phase of the study, and selected for use in the task. The full cultural adaptation procedure is described elsewhere.

#### 2.4.1 Self‐Reports

Three self‐report questionnaires were selected for this study: (a) Measurement of Mental Health among Adolescents and Young People at the Population Level Tool (MMAPP, Carvajal‐Velez et al. 2023), which consists of 28 items rated on a 4‐point Likert scale, ranging from “Never” to “Always.” This tool includes all items from two other questionnaires: the Patient Health Questionnaire modified for adolescents (PHQ‐A; Johnson et al. 2002) and the Generalized Anxiety Disorder Scale (GAD‐7; Spitzer et al. 2006). (b) The Disruptive Behavior International Scale (DBIS; Burkey et al. 2018) includes 8 items on a similar 4‐point scale. (c) The Difficulties in Emotion Regulation Scale (DERS)—Short Form (Kaufman et al. 2016), rated on a 5‐point Likert scale from “Almost Never” to “Almost Always.” We used 15 instead of 18 items in this study by combining two items from the Goals subscale into one and removing two Impulse items that adolescents in the Nepali and Spanish translations found redundant. Cronbach's *α* scores for this scale remained high (Colombia *α* = 0.82, Nepal *α* = 0.79, and South Africa *α* = 0.83).

#### 2.4.2 Neuropsychological Tasks

##### 2.4.2.1 Emotional Go/No‐Go

We adapted an emotional go/no‐go task from Tottenham et al. (2011). The task consisted of four blocks in total, each containing 30 randomly ordered images presented to participants on the tablet. Of these, 20 images were associated with the “go” trials, and 10 corresponded to the “no‐go” trials. The stimuli included country‐specific images of five male and five female adolescents for each facial expression. The experimental blocks were presented in the following order: happy–neutral, sad–neutral, neutral–sad, and neutral–happy combinations. In each block, the first emotion represented the “go” trials, and the second represented the “no‐go” trials. To familiarize participants with the task, 20 initial practice trials of surprise‐neutral pairs were administered with correct/incorrect feedback. Each image was displayed for 500 ms, followed by a fixation cross for 1000 ms (a response timeout of 1500 ms). Participants were instructed to respond to “go” trials by touching anywhere on the tablet screen as quickly and accurately as possible, and to refrain from responding to “no‐go” trials based on the instructions provided at the start of each block. This task has been administered only in Colombia and South Africa, not in Nepal.

##### 2.4.2.2 Delay Discounting

The task consisted of 30 trials that were done on a self‐paced basis. On each trial, participants saw a question with this template: “Would you rather have [a variable amount of money] now, or [a fixed amount of money] in [a variable time]?” The questions were presented randomly. A picture of the corresponding currency value was shown on the screen, with the variable amount—current time (immediate option) on the left and the fixed amount—variable time (delayed option) on the right. Participants made their choice by touching the currency on the screen. The variable times included 2 days, 1 week, 1 month, 6 months, and 1 year. The variable amounts were adjusted for each country's currency as follows: 1, 5, 10, 20, 50, and 100 South African rand; 1000, 2000, 5000, 10,000, 20,000, and 50,000 Colombian pesos; and 5, 10, 20, 50, 100, and 500 Nepalese rupees. The fixed amount was set at twice the highest variable amount.

#### 2.4.3 Heart Rate Variability

Two devices were used for cardiac recording: the Polar H10 measures electrocardiogram (ECG) signals with a sampling rate of 130 Hz, using a single‐lead configuration. It is worn with a chest strap that has two electrodes on its inner surface, placed at the level of the processus xiphoideus, with moisture applied to the electrode sides. The Polar H10 has shown reliable performance in measuring HR and HRV indices in prior validation studies and was the priority choice (Hinde et al. 2021; Speer et al. 2020). For private and confidential application of the chest strap, secure locations were designated to safeguard participants. The HeartMath device was attached to the earlobe using an ear clip and measures photoplethysmography (PPG) using LED photodetectors with a sampling rate of 500 Hz. We have validated a predecessor version of the HeartMath device in a prior study (Sinichi et al. 2024, 2025). The device was tested under various conditions, including a 5‐min seated rest matching the current study's instructions, against a gold‐standard ECG, the Vrije Universiteit Ambulatory Monitoring System (de Geus et al. 1995; Willemsen et al. 1996). It detected 99.74% of heart periods, and the correlation coefficient for log‐transformed RMSSD between the device and ECG in the seated condition was 0.95; therefore, it demonstrated that it provides reliable HR and HRV metrics under resting conditions. The HeartMath device was chosen where local feasibility constraints (e.g., incompatible clothing, lack of appropriate secure space, or parental hesitation about the device) ruled out the use of the chest strap. All recordings from Colombia were done using Polar H10; however, the majority of the recordings in Nepal and South Africa were obtained with the HeartMath device.

The measurement was conducted for a 5‐min resting state according to standard protocols (Laborde et al. 2017; Malik et al. 1996; Shaffer and Ginsberg 2017), with the participant seated, knees at a 90‐degree angle, hands resting on their thighs facing upward, eyes closed, and instructed to breathe normally and spontaneously. Heart periods were detected using the proprietary algorithms of the devices and transmitted via Bluetooth with corresponding timestamps to the HRV Logger Android application (Plews et al. 2017), installed on the tablets.

### 2.5 Data Processing and Feature Extraction

All raw data were exported as .csv files: ODK data from the cloud, neuropsychological task data from the app after completion, and heart period time series from the HRV Logger app locally. For self‐report data, missing values were identified and imputed using multilevel multiple imputation, and key outcome scores (MMAPP, DERS, and DBIS) were computed based on predefined item summations. Neuropsychological task data were preprocessed to extract valid task trials per individual and exclude practice trials, and then false alarm and immediate reward choice percentages were calculated; lastly, heart period time series underwent artifact correction before deriving RMSSD and mean HR for analysis. The pre‐processing and feature extraction of neuropsychological tasks and cardiac data were performed using Python version 3.12.3 in the Visual Studio Code environment. For self‐reports, the process was done in RStudio version 2024.12.0 Build 467.

#### 2.5.1 Self‐Reports

##### 2.5.1.1 Handling Missing Data

Responses categorized as “other,” “don't know,” “prefer not to say,” or empty fields were treated as missing values. The proportion of missing data was computed for each questionnaire and demographic item. Across all countries, missing data were minimal: the mean percentage of missing data for MMAPP, DERS, DBIS, and demographic items ranged from 0% to 1%, with the maximum for any single item ranging from 1% to 3%.

Next, we assessed the mechanism of missingness by examining correlation coefficients between missingness in key outcome variables and potential auxiliary variables (Woods et al. 2021, 2024). The missingness in our items of interest was not strongly correlated with any of the other variables (*r* < 0.1). We proceeded with multilevel multiple imputation using the mice (Multivariate Imputation by Chained Equations) package in R (van Buuren and Groothuis‐Oudshoorn 2011). The imputation model included key outcome variables (MMAPP, DERS, and DBIS items), predictors (log‐transformed RMSSD), covariates (age, gender, height, and weight), and Country as the clustering variable. Imputation methods were assigned based on variable types: proportional odds regression for ordinal variables, logistic regression for nominal variables, and two‐level normal models for continuous variables to account for the clustering effect of Country. Derived variables, such as BMI and total scores, were also imputed passively. We generated five (*m* = 5) imputed datasets over 10 iterations (maxit = 10). After the imputation, we confirmed the convergence of the model by examining the trace plots. The final imputed dataset was exported for subsequent analyses.

##### 2.5.1.2 Calculating Outcome Variables

The MMAPP‐PHQA score was calculated by adding all 9 relevant items/symptoms from the MMAPP. As part of the cultural adaptation, some items were divided into multiple subquestions to improve comprehension. In these cases, the subquestions were treated as belonging to the same symptom domain, and the highest score among them was used to represent that domain. Specifically, one derived item representing negative mood was calculated as the maximum of “Sad or depressed,” “Annoyed or irritable,” and “Hopeless.” A second derived item representing sleeping problems was the maximum of “Difficulty falling asleep,” “Waking up at night,” and “Sleeping too much.” A third derived item representing motor symptoms was the maximum of “Moving slowly” and “Restless or can't sit still.” These three derived items were then combined with the remaining individual items: “Not enjoying,” “Easily tired or no energy,” “Not wanting to eat or eating too much,” “Felt like a failure,” “Trouble concentrating,” and “Rather be dead or hurt yourself.”

The MMAPP‐GAD7 score was calculated as the sum of seven anxiety‐related items: “Feeling nervous or anxious,” “Unable to stop or control worries,” “Worried about different things,” “Difficulty relaxing or feeling calm,” “Restless or can't sit still,” “Annoyed or irritable,” and “Worried bad things will happen.” The MMAPP‐Total score was computed by taking the maximum value of “Difficulty falling asleep,” “Waking up at night,” and “Sleeping too much” and adding it to the sum of all MMAPP items.

For DERS, reverse coding was applied to “Pay attention to how you feel,” “Care about what you are feeling,” and “Aware of your emotions.” The DERS total score was then obtained by summing all 15 items. Finally, the DBIS total score was calculated as the sum of all eight DBIS items.

#### 2.5.2 Neuropsychological Tasks

During task administration, stimuli and responses for each trial were appended line by line to a .csv file on the tablet, which contained data from multiple participants. The files were parsed in multiple steps to extract valid task trials per individual, and practice trials were excluded during preprocessing. After preprocessing, we calculated the percentage of false alarms for each participant in the emotional go/no‐go task. A false alarm was defined as a response made during a “no‐go” trial, where participants were instructed not to respond to a specific emotion. This percentage was calculated by dividing the total number of false alarms by the total number of responses and multiplied by 100. For the delay discounting task, we computed the percentage of immediate reward choices by dividing the total number of selections for the immediate reward option by the total number of responses across all trials and multiplied by 100.

#### 2.5.3 Heart Rate Variability

A self‐developed Python script in combination with the Python package HRV‐Analysis (Champseix et al. 2021) was used to process the heart period timeseries. First, artifacts were identified, removed, and linearly interpolated. A heart period was flagged as a potential artifact if it met any of the following criteria: (a) Any heart period below 300 ms or above 2000 ms. (b) Deviant beats detected using the Karlsson method (Karlsson et al. 2012), which iteratively compares each heart period to the local mean of preceding and subsequent beats. If the current beat deviated from this local mean by a predefined “deviation threshold,” it was considered an artifact.

In the pre‐registration, we set this deviation threshold at 0.35 for all participants. However, during processing, we employed a personalized approach to determine the threshold based on participant baseline variability. This personalized deviation threshold was based on resting‐state HRV norms from a study with a close age cohort (Jarrin et al. 2015). Using the values from this study (mean = 42.9, SD = 13.4), we categorized our participants into HRV groups as follows: very low (−2 SD), low (−1 SD), normal (−1 to +1 SD), high (+1 SD), and very high (+2 SD). The Karlsson method's deviation thresholds were tailored to these categories: 0.50 for very high HRV, 0.45 for high HRV, and 0.40 for all other categories. This approach minimized overcorrection in individuals with inherently high heart period variability (i.e., a higher threshold is assigned to people with higher baseline HRV).

After artifact detection, removal, and interpolation, participants with more than 10 detected artifacts were excluded from further analysis. This threshold of 10 was chosen experimentally, because in most cases, either the signal‐to‐noise ratio was so poor overall that artifacts were present throughout the entire recording, or it was good overall with none or only a few artifacts, so it effectively differentiated these groups. Additionally, recordings with abnormally short durations or implausibly low heartbeat counts over the 5‐min period, which were identified through visual inspection relative to recording length and mean heart rate, were excluded. Following pre‐processing, RMSSD, the number of artifacts, and mean HR were calculated. Due to the right‐skewed distribution of RMSSD, a natural log transformation was applied for subsequent analyses. Moreover, to evaluate whether the resting state was successfully achieved, RMSSD values were recomputed for each minute of the 5‐min recording to see if any sign of initial withdrawal and later stabilization/increase was present; however, the minute‐wise RMSSD means and standard deviations were relatively stable, and therefore no such sign was observed (see the Supporting Information for the analysis). RMSSD and mean HR were computed using the following formulas:

$$\text{RMSSD}=\sqrt{\frac{1}{N-1}\sum_{i=1}^{N-1}{\left({\mathrm{HP}}_{i}-{\mathrm{HP}}_{i-1}\right)}^{2}}$$

where ${\mathrm{HP}}_{i}$ represents the *i*th heart period, and N is the total number of heart periods in 5‐min.

$$\text{Mean}\;\mathrm{HR}=\frac{60,000}{\text{mean}\left(\mathrm{HPs}\right)}$$

where $\text{mean}\left(\mathrm{HPs}\right)$ represents the mean of the heart periods.

### 2.6 Data Analysis

Below the confirmatory analysis (those outlined in pre‐registration) and exploratory analysis (an additional set of equivalence testing and country‐specific analyses) are explained. It is important to mention that as described in Section 2.5.1, self‐report data has gone through multiple imputation, and therefore, the following analytical models have been adapted to fit the imputed dataset. In particular, analyses were performed separately on each imputed dataset, and the results were pooled using Rubin's rules (Rubin 1987) to account for variability between imputations. In all analyses, we used an alpha level of 0.05 to determine statistical significance.

#### 2.6.1 Confirmatory Analysis

For the confirmatory analysis, data were pooled from all countries. We ran seven separate linear mixed models using the lme4 package (Bates et al. 2015) in R (RStudio version 2024.12.0). In all models, the intercept of Country (Nepal, Colombia, South Africa) was treated as the clustering variable, and the log‐transformed RMSSD was included as the predictor. Age, Gender, BMI, and Device Type were controlled as factors and covariates in the model. The models were fitted separately for different outcome variables of interest from neuropsychological and self‐report data. The psychopathology dependent variables included MMAPP‐Total, MMAPP‐PHQA, MMAPP‐GAD7, and DBIS. Self‐regulation outcome measures included DERS, False Alarm Percentage (from the Emotional Go/No‐Go Task), and Immediate Reward Percentage (from the Delay Discounting Task). Residuals were visually inspected using Q‐Q plots and histograms to ensure that the mixed model assumptions were met.

#### 2.6.2 Exploratory Analysis

##### 2.6.2.1 Equivalence Testing

To evaluate whether the effect of log‐transformed RMSSD on our outcome variables of interest is substantial enough to be considered practically meaningful, we conducted equivalence testing as an exploratory analysis (Schuirmann 1987; Lakens 2017; Lakens et al. 2018; Alter and Counsell 2023). We used the same modeling strategy described in the confirmatory analysis section. However, instead of treating the log‐transformed RMSSD as a continuous variable, we dichotomized it into a categorical variable with two levels: High‐HRV and Low‐HRV, based on a median split. This transformation was chosen to simplify interpretation in the subsequent step. As the next step, we set our smallest effect size of interest (SESOI). Although SESOI must be pre‐specified before examining the data, this was not included in our pre‐registration. Nevertheless, we adopted a conservative approach to ensure that our equivalence test results would provide meaningful insights for the scientific community.

We defined our SESOI as a ±5% change in the dependent variable for every unit change in the independent variable. When dichotomized, this translates to considering the effect negligible if the change in dependent variables between Low‐HRV and High‐HRV groups (based on the median split of log‐transformed RMSSD) is less than 5%. This choice of 5% is practical and captures a conservative range. For example, a 5% change corresponds to the following score changes: 1.35 for MMAPP‐PHQA, 1.05 for MMAPP‐GAD7, 3.75 for MMAPP‐Total, 3 for DERS, and 1.20 for DBIS. For PHQ9 and GAD7, prior studies have identified the minimum clinically significant score changes to correspond to meaningful improvements in participants' subjective report: 3 points for PHQ9 (Turkoz et al. 2021) and 4 points for GAD7 (Toussaint et al. 2020). This shows that our chosen SESOI is well below these thresholds.

After defining the SESOI, we derived the effect size (i.e., the mean difference between High‐HRV and Low‐HRV groups) and its standard error from the fitted mixed model. To implement the two one‐sided *t* test (TOST) procedure, we conducted two separate t‐tests: one testing the null hypothesis that the true mean difference is less than the lower bound and the other testing the null hypothesis that the true mean difference is greater than the upper bound. If both *p*‐values from these tests fall below our significance threshold (*p* < 0.05), we conclude that the estimated group difference is small enough that the groups can be considered statistically equivalent. To complement this analysis, we also calculated 90% confidence intervals (CIs) around the estimated effect size. If the entire 90% CI falls within ±5% of the outcome's maximum possible score, the effect is considered small and the groups are deemed equivalent.

##### 2.6.2.2 Nonpooled Country‐Specific Analyses

In addition to the equivalence testing as an exploratory step, we also checked whether the confirmatory findings hold if, instead of pooling data across all countries and using a multi‐level model, we separate the analysis per country. These country‐specific analyses control for potential confounds that are specific to each country, such as differences in altitude, temperature, season, and group administration (in South Africa). To achieve this, for each country, we ran a general linear model with the same predictors (the log‐transformed RMSSD, Age, Gender, BMI, and Device Type) and the same seven outcome variables.

### 2.7 Open Science Practices and Pre‐Registration

The code and data of this study are available from the corresponding author upon request. The pre‐registrations can be found on the Open Science Framework (https://osf.io/m5v6w; https://osf.io/5t9x4).

There are two small deviations from the pre‐registration. First, the pre‐registration states that we use both random slopes and random intercepts in our multi‐level model; only random intercepts were modeled because including random slopes led to nonconvergence of the model. Second, the deviation threshold for pre‐processing of the heart period time series that we pre‐registered was stated to be 0.35. However, we used a dynamic approach to incrementally adjust this for participants with inherently higher HRV, within the range of 0.40 to 0.50. The RMSSD calculated with these two approaches is essentially identical to the pre‐registered approach (*r* = 0.99 in all countries) and does not alter the results, but we believed this to be a more accurate approach rather than applying a single threshold to all participants.

### 2.8 Use of AI‐Generated Content (AIGC) and Tools

The only use of AI tools in the current paper is limited to improving grammar and syntax in writing, as well as assisting in code development and debugging. No content is generated by AI.

## 3 Results

### 3.1 Descriptive Statistics

For HRV data collection, 100% of participants in Colombia, 34.95% in Nepal, and 22.33% in South Africa used the Polar H10 chest strap, and the rest used the earlobe PPG HeartMath device. As mentioned, the total number of participants included for analysis in the current study was 1107. It is important to note that this refers to the data for which HRV remained available after preprocessing. The initial pool included 1344 participants, from which 5.65% in Colombia, 6.29% in Nepal, and 37.03% in South Africa were excluded, as their cardiac data did not meet the quality criteria specified in Section 2.5.3. From the 1107 participants whose HRV data were available, a portion of the data was lost during preprocessing due to technical issues. The resulting data loss percentages per task and country were as follows: for EGNG, 5.06% in Colombia and 11.63% in South Africa; and for DDT, 0.48% in Colombia, 0.54% in Nepal, and 13.84% in South Africa. For missing data in self‐reports, see Section 2.5.1.

Table 1 shows the descriptive statistics for demographics, heart rate variability, self‐regulation, and psychopathology across the three study sites. Chi‐square tests for gender and age were significant at *p* < 0.001, and ANOVAs for all the other variables listed in the table (all *p* < 0.001; Immediate Reward Percentage, *p* < 0.01) showed significant differences across countries. Gender distribution varied, with a higher proportion of female participants in South Africa (68.87%) compared to Colombia (52.76%) and Nepal (52.15%), in the selected sample. Height and weight showed differences between sites: participants in Nepal were on average shorter (*M* = 1.56 m, SD = 0.07) and weighed less (*M* = 46.96 kg, SD = 8.12) than those in Colombia (*M* = 1.60 m, SD = 0.08; *M* = 52.16 kg, SD = 8.73) and South Africa (*M* = 1.64 m, SD = 0.11; *M* = 56.12 kg, SD = 14.86). BMI was more comparable across countries, ranging from 19.32 (SD = 2.77) in Nepal to 20.84 (SD = 5.53) in South Africa.

**TABLE 1:** Descriptive statistics for demographics, self‐reports, neuropsychological measures, and heart rate variability.

Outcome	Range (Tukey‐fence)	Colombia	Nepal	South Africa
Demographics				
Male	—	46.24% (2.80)	47.85% (2.80)	31.13% (2.60)
Female	—	52.76% (2.81)	52.15% (2.80)	68.87% (2.60)
Nonbinary	—	1.01% (0.57)	—	—
Age	13.00–15.00	13.83 (0.79)	13.87 (0.74)	14.21 (0.72)
Height (m)	1.35–1.85	1.60 (0.08)	1.56 (0.07)	1.64 (0.11)
Weight (kg)	26.00–74.70	52.16 (8.73)	46.96 (8.12)	56.12 (14.86)
BMI (kg/m^2^)	11.45–28.05	20.35 (2.95)	19.32 (2.77)	20.84 (5.53)
Heart rate (variability)				
RMSSD (ms)	5.18–109.71	47.54 (31.81)	42.28 (21.27)	59.92 (28.72)
Mean HR (bpm)	54.02–119.60	84.73 (12.87)	89.82 (11.71)	84.00 (11.69)
Self‐regulation domain				
DERS	0.00–50.00	24.61 (10.05)	22.72 (8.97)	26.77 (10.60)
False alarm percentage	0.00–32.50	14.17 (6.92)	—	15.97 (6.13)
Immediate reward percentage	0.00–100.00	37.76 (24.78)	42.03 (29.29)	44.56 (22.76)
Psychopathology domain				
MMAPP‐GAD7	0.00–19.00	8.20 (4.35)	6.18 (3.83)	7.62 (4.11)
MMAPP‐PHQ9	0.00–23.00	10.36 (5.62)	8.60 (4.75)	10.10 (5.14)
MMAPP‐Total	0.00–59.00	25.26 (14.24)	20.83 (11.80)	25.10 (12.91)
DBIS	0.00–13.00	6.47 (3.43)	3.97 (2.86)	5.81 (3.77)

*Note:* Demographics and self‐report measures underwent multiple imputation and are presented as pooled estimates according to Rubin's rules. Continuous variables are reported as Mean (Standard Deviation), and gender distribution is presented as Percentage (Standard Error). Demographic indicators include gender, age (years), height (meters), weight (kilograms), and body mass index (BMI). Physiological measures include RMSSD (Root‐Mean‐Square of Successive Differences, in milliseconds) and Mean HR (Mean Heart Rate, in beats per minute). Self‐regulation outcomes include DERS (Difficulties in Emotion Regulation Scale–Short Form), false alarm percentage from the Emotional Go/No‐Go (EGNG) task, and immediate reward percentage from the Delay Discounting Task (DDT). Psychopathology outcomes include MMAPP‐GAD7 (Generalized Anxiety Disorder), MMAPP‐PHQA (Patient Health Questionnaire for Adolescents), MMAPP‐Total (total symptom score), and DBIS (Disruptive Behavior International Scale). The EGNG task was not administered in Nepal. The Range (Tukey‐fence) column reflects the interquartile‐based Tukey fence range that excludes approximately 0.7% of the outliers in a normally distributed variable.

Resting‐state RMSSD showed a notable difference between countries, with Colombia (*M* = 47.54, SD = 31.81) and Nepal (*M* = 42.28, SD = 21.27) being more similar, while South Africa had higher HRV (*M* = 59.92, SD = 28.72). Mean HR was relatively stable across countries, with only a difference of about five beats per minute (ranging from *M* = 84.00 in South Africa to *M* = 89.82 in Nepal). In the self‐regulation and psychopathology domain, the changes in scores between countries ranged from approximately 2% to 11% of the maximum possible scores. DERS differed by about 7% between countries; Nepal had the lowest mean (*M* = 22.72), while South Africa had the highest (*M* = 26.77), with Colombia in between. Immediate Reward Percentage showed a similar pattern. False Alarm Percentage also showed similarity in Colombia and South Africa, with about a 2% score difference (the EGNG was not administered in Nepal). In the psychopathology domain, anxiety, depression, and total symptom scores varied modestly (~6%–10%). Anxiety and depression scores were slightly higher in Colombia and South Africa than in Nepal. Finally, in externalizing symptoms (DBIS), Nepal had a notably lower mean (*M* = 3.97) than South Africa (*M* = 5.81) and Colombia (*M* = 6.47).

#### 3.1.1 Ethnic Composition

In terms of ethnic composition, in Colombia (*n* = 417), 9.83% identified as Negro, Mulatto, Afro‐descendant, or Afro‐Colombian, followed by smaller proportions identifying as Indigenous (4.56%), Gypsy or Roma (0.96%), Palenquero of San Basilio (0.48%), and Raizal of the Archipelago of San Andres, Providencia and Santa Catalina (0.24%), while the majority (70.98%) reported none of the listed ethnic categories. In Nepal (*n* = 372), most participants identified as Janajati (49.73%) or Brahmin/Chhetri (28.76%), with smaller proportions identifying as Dalit (9.68%), Madhesi (8.6%), or Muslim (2.96%). In South Africa (*n* = 318), the majority identified as Black (83.96%) and the remainder as Colored (16.04%).

### 3.2 Confirmatory Analysis

Seven linear mixed models were fitted to examine the relationship between log‐transformed RMSSD and the outcome variables of interest (similar findings were replicated when HF‐HRV was used as the predictor, see the Supporting Information). Table 2 presents the regression coefficients for log‐transformed RMSSD as the predictor, while controlling for the effects of Age, BMI, Gender, and Device Type. The reported *B* coefficients represent unstandardized estimates for the effect of log‐transformed RMSSD on the respective outcome variables. See Supporting Information for full regression tables that include all factors and covariates.

**TABLE 2:** Regression table for the effect of log‐transformed RMSSD on outcome variables.

Outcome	*B*	SE	*t*	df	*p*	*R* ^2^ (Conditional)	ICC
Self‐regulation domain							
DERS	0.74	0.51	1.44	1091.49	0.15	0.13	0.02
False alarm percentage	−0.06	0.46	−0.13	661.99	0.898	0.07	0.05
Immediate reward percentage	0.54	1.46	0.37	933.89	0.713	0.01	0.01
Psychopathology domain							
MMAPP‐GAD7	0.41	0.22	1.87	1089.31	0.061	0.13	0.05
MMAPP‐PHQ9	0.24	0.28	0.86	1086.97	0.39	0.11	0.03
MMAPP‐Total	0.96	0.68	1.41	1090.45	0.16	0.13	0.03
DBIS	0.63	0.18	3.4	1082.03	**< 0.001**	0.14	0.12

*Note:* The table presents the unstandardized regression coefficients (*B*) from linear mixed models, examining the effect of log‐transformed RMSSD (Root Mean Square of Successive Differences) on seven pre‐registered outcome variables. All models control for Age, Gender, Body Mass Index (BMI), and Device Type (PPG/ECG) with Country modeled as a random intercept. Outcome variables are grouped by domain: Self‐regulation (DERS: Difficulties in Emotion Regulation Scale–Short Form; False Alarm Percentage from the Emotional Go/No‐Go Task; Immediate Reward Percentage from the Delay Discounting Task), and psychopathology (MMAPP‐GAD7: Generalized Anxiety Disorder; MMAPP‐PHQA: Patient Health Questionnaire–Adolescent version; MMAPP‐Total: Total symptom score; DBIS: Disruptive Behavior International Scale). SE = Standard Error of the estimate; *t* = test statistic for the *B* coefficient; df = degrees of freedom. *R* ^2^ (Conditional) reflects the proportion of variance explained by both fixed and random effects. ICC = Intraclass Correlation Coefficient, indicating variance attributable to between‐country differences. The bold value for DBIS indicates a statistically significant association (p < 0.001).

Of the seven models, six indicated a nonsignificant relationship between log‐transformed RMSSD and the outcome variables. The only exception was the model predicting DBIS scores, which demonstrated a significant effect, *R* ^2^ = 0.14, *t*(1082.03) = 3.4, *p* < 0.001, suggesting that higher HRV values were associated with higher DBIS scores. Given that seven tests were performed, this effect still remains significant after Bonferroni correction (*p* = 0.005). For the remaining models, conditional *R* ^2^ values ranged from 0.01 to 0.13, and the intraclass correlation coefficients (ICCs) of the mixed models ranged from 0.01 to 0.05.

**FIGURE 1:**
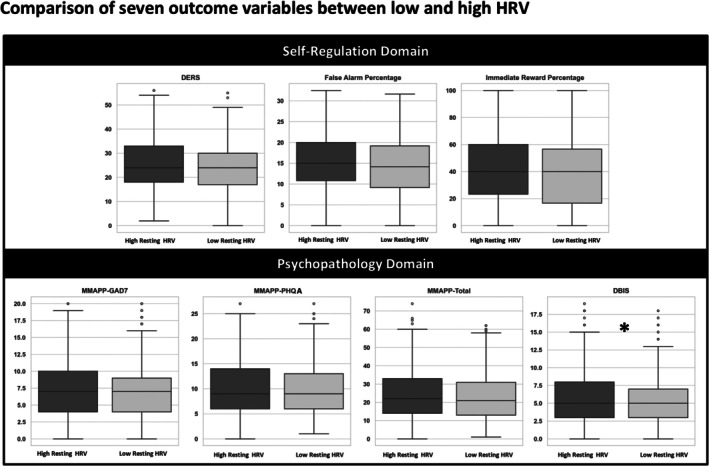
Comparison of seven outcome variables between low and high resting‐state HRV. Overview of the seven outcome variables based on pooled data, stratified by resting‐state HRV. Each subplot corresponds to one outcome variable within either the self‐regulation or psychopathology domain. HRV groups were defined using a median split of log‐transformed RMSSD (Root Mean Square of Successive Differences) values. The *x*‐axis reflects HRV group (low vs. high), and the *y*‐axis presents raw scores for DERS (Difficulties in Emotion Regulation Scale–Short Form), MMAPP‐GAD7 (Generalized Anxiety Disorder), MMAPP‐PHQA (Patient Health Questionnaire for Adolescents), MMAPP‐Total (total symptom score), and DBIS (Disruptive Behavior International Scale), as well as percentages for the neuropsychological outcomes: False alarms (Emotional Go/No‐Go Task) and immediate reward choices (Delay Discounting Task). An asterisk (*) denotes the only statistically significant effect observed in the confirmatory multilevel model analysis.

Figure 1 shows the descriptive overview of each outcome variable from the pooled data, stratified by a median split of log‐transformed RMSSD on the *x*‐axis. The only significant effect (DBIS) is indicated with an asterisk (*) on the plot. As the figure shows, the distribution characteristics of all outcome variables are similar for both low HRV and high HRV groups.

### 3.3 Exploratory Analysis

The results of the equivalence testing using both the TOST procedure and the 90% confidence interval comparison are summarized in Table 3. For all outcome variables in both the self‐regulation and psychopathology domains, the difference between the low‐HRV and high‐HRV groups was negligible, based on the equivalence bounds of ±5%. In other words, any differences in the outcome variables, are less extreme than the equivalence range between the low and high HRV groups. This is evident from all significant lower and higher *p*‐values of the one‐sided t‐tests against the lower and upper bounds for each outcome variable. Additionally, for all outcome variables, the 90% confidence intervals fall entirely within the equivalence bounds.

**TABLE 3:** Equivalence testing results for outcome variables.

Outcome	*B*	90% CI	SE	df	ΔL, ΔU	tL	tU	pL	pU
Self‐regulation domain									
DERS	1.62	[0.67, 2.57]	0.58	1090.52	−3.0, 3.0	8	−2.4	**< 0.001**	**0.008**
False alarm percentage	0.14	[−0.73, 1.02]	0.53	661.38	−5.0, 5.0	9.67	−9.13	**< 0.001**	**< 0.001**
Immediate reward percentage	2.01	[−0.7, 4.71]	1.64	982.36	−5.0, 5.0	4.26	−1.82	**< 0.001**	**0.034**
Psychopathology domain									
MMAPP‐GAD7	0.52	[0.12, 0.93]	0.25	1086.24	−1.05, 1.05	6.4	−2.14	**< 0.001**	**0.016**
MMAPP‐PHQ9	0.52	[0.01, 1.03]	0.31	1088.16	−1.35, 1.35	6.04	−2.67	**< 0.001**	**0.004**
MMAPP‐Total	1.6	[0.34, 2.87]	0.77	1088.58	−3.75, 3.75	6.96	−2.79	**< 0.001**	**0.003**
DBIS	0.64	[0.3, 0.98]	0.21	1082.09	−1.2, 1.2	8.89	−2.69	**< 0.001**	**0.004**

*Note:* The table presents results from equivalence testing comparing outcome variables between low‐HRV and high‐HRV groups, based on a median split of log‐transformed RMSSD (Root Mean Square of Successive Differences). Outcome variables span self‐regulation (DERS: Difficulties in Emotion Regulation Scale–Short Form; False Alarm Percentage from the Emotional Go/No‐Go Task; Immediate Reward Percentage from the Delay Discounting Task) and psychopathology (MMAPP‐GAD7: Generalized Anxiety Disorder; MMAPP‐PHQA: Patient Health Questionnaire for Adolescents; MMAPP‐Total: Total symptom score; DBIS: Disruptive Behavior International Scale). *B* = unstandardized regression coefficient representing the mean group difference; 90% CI = confidence interval for the observed difference; SE = standard error of the estimate; df = degrees of freedom; ΔL, ΔU = lower and upper bounds of the smallest effect size of interest (SESOI); tL, tU = test statistics from the two one‐sided *t*‐tests (TOST); pL, pU = corresponding *p*‐values. Equivalence is concluded when both pL and pU fall below the *α* = 0.05 threshold, and the 90% CI lies fully within the defined equivalence bounds. Bold values indicate statistically significant results (p < 0.05) for both one‐sided tests, confirming equivalence.

As a further exploratory analysis, we conducted separate general linear models for each country instead of pooling data. These regression tables can be found in the Supporting Information. In short, after controlling for the family‐wise error rate through Bonferroni correction, given that for Colombia and South Africa 7 tests and for Nepal 6 tests were performed, none of these *p*‐values remain significant. Hence, the results are similar to the findings from the multi‐level model in the confirmatory pooled data analysis.

## 4 Discussion

The current study examined whether resting‐state HRV is associated with self‐regulation and psychopathology in a cross‐sectional sample of adolescents living in poverty across a large, multi‐continental, and diverse sample from Colombia, Nepal, and South Africa. We tested seven pre‐registered hypotheses, based on the overall assumption that lower HRV is associated with weaker self‐regulatory mechanisms (lower inhibitory control, lower delay discounting, and lower emotional regulation) and higher psychopathology symptoms (depression, anxiety, and externalizing symptoms).

Across all models, HRV was not significantly associated with any of the outcomes, with the exception of a small effect on externalizing behavior in the opposite direction of our expectation. Other studies have also shown mixed results regarding resting‐state HRV and externalizing symptoms, ranging from a study that found lower resting‐state HRV to be associated with both internalizing and externalizing symptoms in urban boys aged 7–11 from impoverished neighborhoods (Pine et al. 1998), to work by Panteli et al. (2024) that found children with externalizing behavior showed higher resting‐state HRV compared to the control group (though only significant for SDNN and not RMSSD). In a third study, resting HRV and HRV reactivity in adolescents (13–17 years old) interacted with childhood adversity to predict internalizing, but not externalizing symptoms (McLaughlin et al. 2014). Taken together, our opposite‐direction estimate for externalizing symptoms, alongside mixed prior results, most likely reflects a context‐ and method‐dependent signal rather than a stable association. Therefore, we next tested whether this externalizing finding and the other estimates are practically meaningful using equivalence testing.

Accordingly, we complemented our findings with equivalence testing to assess whether the effect sizes were practically meaningful and found that all effect sizes were too small and the groups were statistically equivalent. In other words, whether adolescents had high or low resting‐state HRV did not meaningfully differentiate them on any of the seven outcome variables related to either self‐regulatory mechanisms or psychopathology symptoms. This also applied to the only significant association we initially found, where we rejected any effects larger than the prespecified SESOI (±1.20 score on the DBIS with a 5% error rate). That means, if a true effect exists between the low HRV and high HRV group, the difference on the DBIS questionnaire between the groups is smaller than this value, and hence we consider it negligible.

The lack of evidence for an association between resting‐state HRV and these domains stands in contrast to the majority of the existing literature, which has demonstrated such relationships in meta‐analyses and systematic reviews examining HRV and self‐regulatory mechanisms (Forte et al. 2019; Holzman and Bridgett 2017; Nicolini et al. 2024; Zahn et al. 2016) as well as psychopathology (Brown et al. 2018; Chalmers et al. 2014; Cheng et al. 2022; Ding et al. 2024; Ramesh et al. 2023). These findings also challenge theoretical frameworks like the neurovisceral integration (NVI) model, which hypothesizes the universal existence of these associations, though it is noteworthy that more recent accounts of this model are embracing a sociocultural perspective regarding the expected directionality of the effects (Watanabe, Kitayama, et al. 2025; Watanabe, Tyra, et al. 2025). Although the current design does not allow for a deeper investigation into the cause of this absence of effect, we speculate on a few possible explanations based on the literature in the following sections.

### 4.1 Age and Autonomic Nervous System Developmental Trajectory

A notable trend in our sample of adolescents aged 13–15 is that HRV was higher in South Africa, where the majority of the sample identified as Black (Mean RMSSD = 59.92 ms), a pattern previously observed in African American samples (Hill et al. 2015; Watanabe, Tyra, et al. 2025). The source of this difference remains unclear, in part because of a lack of evidence for ethnic variation in heritability estimates (Wang et al. 2005). However, some researchers have speculated that it may reflect a compensatory regulatory strategy shaped by experiences of racism and other sociocultural stressors (Watanabe, Kitayama, et al. 2025).

Additionally, compared to other studies, HRV in our sample appears to be slightly lower (Colombia 47.54 ms; Nepal 42.28 ms) than typical ranges reported for age‐matched groups, though methodological differences (e.g., recording duration and setting) may limit direct comparisons. For instance, Harteveld et al. (2021) reported median RMSSD values for ages 13–15 of 56.7 ms (boys) and 45.6 ms (girls) using 2–10 min resting‐state data; Tegegne et al. (2020) found medians for ages 13–14 of 67.4 ms (boys) and 66.5 ms (girls) from 10 s ECGs; and Silvetti et al. (2001) reported means for ages 11–15 of 71 ms (boys) and 77 ms (girls) using 24 h Holter monitoring. It is known that HRV peaks in early adolescence and usually declines thereafter (Quigley et al. 2024). Different aspects of executive function mature at different developmental stages (Igazság et al. 2019), with inhibitory control in particular continuing to develop into early adulthood, where it typically surpasses adolescent levels (Ferguson et al. 2021). Furthermore, adolescence also marks the onset of most psychopathology symptoms (Paus et al. 2008).

Keeping with this theme, two interesting findings come from Holzman and Bridgett's (2017) meta‐analysis on HRV and self‐regulation. First, older individuals exhibited a stronger relationship between HRV and self‐regulation, and second, that the only age group in the meta‐analysis for which no significant association between HRV and self‐regulation was observed was adolescents aged 13–17; the age range of our current sample. This could theoretically be linked to the ongoing development of prefrontal cortical structures during this period, but it may also be a statistical power issue due to the small number of included studies in the meta‐analysis for this age group, as noted by the authors. Further evidence for a potential developmental difference in adolescence comes from Koenig et al. (2018), who found that, unlike in adults, cortical thickness and HRV were inversely correlated in adolescents, with higher cortical thickness associated with lower HRV. Similarly, Wesarg et al. (2022) in their meta‐analysis on childhood adversity and vagal reactivity, found that older individuals showed a stronger link between past adversity and blunted vagal reactivity, suggesting that such physiological patterns may accumulate or become more apparent with age. Relatedly, a meta‐analysis of HRV and depression in children and adolescents showed that the correlation between HF‐HRV and depression was stronger in those aged 12 years and older (Ding et al. 2024).

Overall, these findings may suggest a specific developmental trajectory for the autonomic and central nervous systems, and a weaker cortical–autonomic coupling during adolescence that may hinder our ability to measure the relationship between HRV, self‐regulation, and psychopathology, which requires further investigation.

### 4.2 Socioeconomic Status, Adversity, and Ethnic Differences

Several studies have highlighted the impact of socioeconomic adversity on autonomic nervous system dysregulation (Busso et al. 2017; Gruenewald et al. 2012; Voellmin et al. 2015; Wesarg et al. 2022). For example, greater perceived lifetime discrimination has been shown to predict lower resting HRV in African American samples (Hill et al. 2017), and reduced HRV was suggested to be a potential moderator of early life adversities in general and the development of psychopathology (Sigrist et al. 2021). The current sample consists of adolescents living in high‐poverty, disadvantaged areas, with diverse ethnic backgrounds, making it distinctly different from most published research, which focuses on more socioeconomically advantaged or predominantly White populations. As such, one cannot assume that findings from western, educated, industrialized, rich, and democratic populations will replicate identically in other contexts such as the LMIC in this study before the assumption of universality has been thoroughly tested. Besides the current study, an increasing body of other evidence suggests more nuanced and context‐dependent rather than universal relationships.

Sturge‐Apple et al. (2016) using a delay‐of‐gratification task found that higher resting‐state HRV was associated with longer delay in children from higher‐SES backgrounds, but this relationship was completely reversed in those from lower‐SES backgrounds. More recently, Tan et al. (2024) showed that subjective socioeconomic status moderated the relationship between resting HRV and pain perception, where higher HRV predicted greater pain perception among young adults with lower subjective SES, which the authors interpret as a pattern of affective upregulation oriented toward heightened threat detection. Interestingly, in a longitudinal study, Barry et al. (2022) found that adolescents from lower socioeconomic backgrounds who had a combination of high heart rate reactivity and poor inhibitory control (on a Stroop task) at age 16 were more likely to develop externalizing behavior symptoms in adulthood, a pattern not observed in higher SES groups. Despite the importance of these findings, and the possibility that HRV‐related mechanisms in low‐SES groups are not necessarily equivalent to those in high‐SES groups, this area remains underexplored. For instance, in their meta‐analytic review on HRV and self‐regulation Holzman and Bridgett (2017) reported that only 19 out of 123 studies included samples with sociodemographic risk factors, while most did not report the sociodemographic characteristics of their participants.

Other factors beyond SES may also contribute to unexpected findings in ethnic groups underrepresented in psychophysiology research. Jennings et al. (2015) showed that, unlike Caucasian American adults, African Americans demonstrated no significant association between resting‐state HF‐HRV, cerebral blood flow, and executive functions, and in some cases, even showed opposite trends. Similarly, Thayer and Koenig (2019) found that the relationship between resting cerebral blood flow in the anterior cingulate cortex and resting‐state HRV was positively correlated in European American adults but negatively correlated in African Americans. Furthermore, emotion regulation strategies showed ethnicity‐specific associations: among African Americans, greater habitual use of reappraisal and anger suppression was linked to higher HRV, while anger expression was associated with lower HRV. These patterns were not observed in European American participants. Both these studies corrected for SES via educational attainment, but it remains to be explored whether the observed ethnic differences reflect social factors (e.g., discrimination, minority stress) or actual biological differences (e.g., physiological variation).

Importantly, a recent work investigating the relationship between resting‐state HRV and self‐regulation in a sample of young adults with Asian (AS), African American (AfAm), Hispanic/Latino (HL), and non‐Hispanic White (NHW) backgrounds showed that the association is moderated by ethnicity (Watanabe, Tyra, et al. 2025). The authors reported that higher HRV was marginally associated with greater reappraisal in AS participants, significantly associated with lower reappraisal in AfAm participants, not significantly associated with reappraisal in HL or NHW participants, marginally associated with lower suppression in NHW participants, and not significantly associated with suppression in the other ethnic groups. The authors highlighted the importance of considering sociocultural contexts, including discrimination, as potential mediators in future studies.

Relative to samples from European descent populations, or high‐income countries, individuals from LMIC settings or with different ethnic backgrounds are significantly underrepresented in the literature. As a result, our understanding of these associations, and the generalizability of theoretical models like the NVI, remains limited and requires further exploration.

### 4.3 Complexity, Specificity, and Methodology

In the explanation of our null findings, we want to bring forward a few clarifications regarding the complexity of interpretation, specificity, and methodological sensitivity of HRV. We also want to stress that similar null findings were already present in the literature and will review some of them briefly.

The first consideration is the complexity inherent in interpreting HRV as a proxy for parasympathetic activity. Several theoretical frameworks, including the NVI model, are premised on the idea that resting‐state HRV serves as an “index” or marker of “vagal tone,” or more broadly (and often overgeneralized), as an indicator of top‐down cortical regulation. This assumption forms the foundation for linking HRV to a wide range of psychological phenomena. However, multiple authors (Grossman 2024; Karemaker 2020; Ritz 2024b), including the recent expert recommendation (Menuet et al. 2025), have criticized this interpretation, specifically the view that HRV is a direct measure of total cardiac vagal activity or overall vagal tone. The observed relationships between HRV metrics and psychological outcomes (assuming they hold up under replication across diverse contexts and populations) may be understood not necessarily as evidence of autonomic nervous system modulation, but through alternative conceptual lenses (see Grossman 2024 for a comprehensive discussion, as well as the special issue in Biological Psychology on the Contributions of the Vagus to Psychological Functioning and Health).

The second caution concerns the methodological choices. Given the degrees of freedom researchers have in selecting and pre‐processing HRV metrics, direct comparisons of study findings may be challenging, and the replicability of some findings may be specific to the particular method used to estimate RSA, designs or processing approaches. For example, how respiration is handled can introduce significant variability and even alter findings (Grossman et al. 1991; Ritz 2024a, 2024b). In the case of phobia and anxiety, differences in HRV findings may be attributable to the hyperventilation commonly observed in these individuals (Ritz 2009). Similarly, associations between HRV and anxiety disorders have been shown to disappear after controlling for medication use (Licht et al. 2009). Moreover, how artifacts are handled and cleaned can have a huge impact on HRV metrics and may lead to overestimation (Berntson and Stowell 1998). In addition to methodological considerations regarding HRV itself, the selection and operationalization of other outcome variables used as correlates (e.g., measures of psychopathology or self‐regulation) are also influenced by researcher choices, further contributing to mixed findings and complicating our understanding. A recent review by McIntyre and Sloan (2025) critically discusses these points in more depth.

Last but not least, null findings like those in the current study need to be taken into account in the accumulation of knowledge. As McGinley et al. (2018) note in a SPR symposium “Escaping the File Drawer: Is Resting Heart Rate Variability Always Useful as a Biomarker for Self‐Regulation?”, an increasing number of findings challenge the widely held assumptions about consistent and universal relationships between HRV, self‐regulation, and psychopathology. Here we will only do a selective review of a few null findings that have similarities with our study, to illustrate these emerging patterns:

Some of the earliest work in this area already included inconsistencies. For instance, Yeragani et al. (1991) reported no standing HRV differences between young adults with depression and healthy controls. Similarly, Dishman et al. (2000) found no association between trait anxiety and any resting‐state supine HRV indices in adults. Frazier et al. (2004) reported no relationship between tonic (resting‐state) HRV and any measures of emotion regulation—subjective, expressive, or physiological—in young adults, and Duschek et al. (2009) found resting HRV unrelated to attentional performance in young adults. More recent studies have continued to report similar null findings. A large and diverse sample of middle‐aged adults from the MIDUS study did not find any association between resting HRV and global executive functions (Kimhy et al. 2013). Moreover, in a sample of adult male U.S. Army personnel (Spangler et al. 2018), resting‐state HRV was unrelated to differences in correct rejections, a measure of inhibitory control, across low‐ and high‐stress conditions. In another study using a go/no‐go task and fNIRS to measure brain activity during inhibition, alongside simultaneous cardiac recordings in adults (Condy et al. 2020), no significant association was found between resting‐state HRV and resting prefrontal activity at the baseline measurement (after correcting for multiple comparisons). Additionally, they found no significant relationship between concurrent HRV and prefrontal activation during the go/no‐go task. Similarly, another study using a cognitive reappraisal task during fMRI found no significant association between task‐related concurrent HRV and mPFC–amygdala connectivity in older adults, and found a significant association in younger adults; however, in an unexpected direction, such that higher task‐related HRV predicted weaker mPFC–amygdala connectivity (Tupitsa et al. 2023). Notably, however, a previous study by Sakaki et al. (2016) using resting‐state HRV, rather than state‐dependent HRV, found an association between resting‐state HRV and right amygdala–mPFC connectivity during resting‐state fMRI in both young and older adults, such that higher HRV was associated with stronger connectivity, which is noteworthy and in line with earlier points about methodological specificity. Another study in 5‐year‐old children who performed a delay of gratification task likewise found no relationship between resting‐state HRV and task performance; however, task‐related HRV changes moderated the link between self‐control strategy class and the ability to wait for the reward (Raghunathan et al. 2022). A recent study using the Short Self‐Regulation Questionnaire (SSRQ) in young adults found no relationship between self‐regulation and resting‐state HRV (Carroll 2025). Moreover, it is noteworthy that the recent study (Watanabe, Tyra, et al. 2025), which advances a “sociocultural” neurovisceral integration model, found no significant associations between resting HF‐HRV and habitual use of suppression or reappraisal in any ethnic group, including non‐Hispanic White participants, where a positive HRV–reappraisal link might most conventionally be expected; the only exception was among African American participants, where higher resting HRV predicted lower reappraisal.

Finally, a new umbrella review of 71 meta‐analyses provides insights that align with the above discussion around complexity, methodological considerations, contextual variables, and emerging null findings in HRV and mental health research (Wang et al. 2025). The review concluded that most reported associations across the pooled analyses on reduced HRV in mental health were supported by only weak or nonsignificant evidence, with no results classified as convincing or highly suggestive. Importantly, the evidence for major depressive disorder and generalized anxiety disorder was even weaker, falling below the threshold for suggestive evidence. The review covered 19 mental disorders including depression and anxiety, PTSD, dementia, schizophrenia, bipolar disorder, and autism spectrum disorder. Finally, no two disorders showed identical patterns of HRV alteration.

While the associations between HRV and psychopathology and self‐regulatory mechanisms remain central to the predictions of the NVI model, and several studies have found such associations, the lack of meaningful relationships in the current work, alongside the growing number of null or unexpected findings in the literature, warrants further attention and investigation. As reviewed above, there is considerable heterogeneity across studies in methodological choices, sampling strategies, and contextual factors, which makes it difficult to draw firm conclusions about the sources of discrepancies in findings (Garrett‐Ruffin et al. 2021). Replication studies with greater attention to these factors are needed to uncover the underlying causes and, equally important, to establish the true effect sizes that have so far been largely overlooked. Using equivalence testing in addition to standard null‐hypothesis significance testing will be especially useful in this regard, as it allows follow‐up research to build on clearer expectations about the magnitude of effects rather than only their presence or absence.

### 4.4 Limitations, Challenges, and Future Directions

The findings of this study need to be interpreted in light of its limitations, most of which arise from the practical challenges of conducting research in real‐world settings that differ significantly from controlled lab environments. One of the key limitations of the current study is the inability to control for respiration parameters, as recommended by several authors (Grossman et al. 1991; Ritz 2024a). This was primarily due to feasibility constraints, which also led to a second limitation: the inability to use a gold‐standard device consistently across all HRV measurements. While all HRV data in Colombia were collected using the Polar H10 chest strap, the majority of the data in Nepal and South Africa relied on a PPG device. Although this device was validated in our previous study (Sinichi et al. 2025), and should perform reliably under resting‐state conditions, we believe that part of the high HRV data loss observed in South Africa (37%) may be attributable to the interaction between PPG sensor performance and melanin concentration in the skin (Hill et al. 2015; Shcherbina et al. 2017), especially since the majority of the sample in South Africa self‐identified as Black (see Section 3.1.1). In the absence of raw ECG/PPG signals, it is ultimately impossible to determine whether the higher HRV observed in the South Africa sample is purely physiologically driven or whether it is, to some extent, the result of misidentified beats that artificially increase HRV values. Nonetheless, we applied a conservative approach to ensure that only clean, artifact‐corrected data were retained for analysis.

A third limitation relates to HRV data collection in school environments. Although research assistants followed standard protocols closely, the school setting may have introduced confounding factors such as agitation, physical activity prior to measurement, caffeine intake, and others. Questions to assess and control these variables were not included, nor was information on sleep/wake cycles, medication use, or cardiac conditions gathered, though the latter two are less likely to significantly affect this age group (13–15 years). Finally, beyond the limitations tied to our independent variable, the interpretation of the dependent variables also comes with its own caveats. Although self‐report tools and neuropsychological tasks were culturally adapted and psychometrically evaluated at each site, many participants in these contexts were likely encountering them for the first time. Unfamiliarity, novelty, or even fatigue and disengagement from the lengthy assessment may have influenced responses and introduced extra noise into the data. Moreover, other studies that reported an association between resting‐state HRV and DERS (e.g., Williams et al. 2015) did so on different facets of emotion regulation and subscales of the DERS, whereas we were limited to reporting total score as the subscales could not be computed due to the removal of some items. Finally, the emotional faces used in the current EGNG task were limited to sad, happy, and neutral; however, including a broader range of emotions specifically aligned with the NVI (e.g., fearful expressions) would extend the operationalization of our construct of interest.

These challenges highlight the importance of improving our understanding of psychophysiology in disadvantaged populations, not only by including more diverse samples and refining theoretical models but also by developing new methodological approaches. Although comprehensive manuals exist for HR and HRV data collection and processing (Laborde et al. 2017; Malik et al. 1996; Quigley et al. 2024), field‐based research presents unique challenges that require rethinking standard procedures. For example, in the absence of raw ECG or PPG waveforms, special attention must be paid to how heart period time series are handled to minimize noise and bias. Likewise, different types of efforts should be made to account for respiration, for instance through paced breathing or frequency‐based techniques (e.g., Thayer et al. 2002). Cultural constraints further complicate research in certain settings. In Nepal, for instance, we encountered substantial resistance to using chest straps, especially among female participants, due to privacy concerns raised by both students and their parents. Future research needs to acknowledge these constraints and prioritize the development of inclusive, scalable, and user‐friendly protocols and tools that make psychophysiological research more feasible in disadvantaged areas. Doing so is essential if we hope to better understand the psychophysiology of the world's majority population.

#### 4.4.1 Conclusions

The current study found no evidence in the theorized direction of an association between resting‐state heart rate variability and indices of self‐regulation or psychopathology in a unique sample of adolescents living in high‐poverty contexts across Colombia, Nepal, and South Africa. Replication studies are needed to confirm these findings. Without overinterpreting the results, we hope this study contributes to a broader rethinking of heart rate variability as a psychophysiological index, particularly in underrepresented populations that have often been assumed to show consistent links between HRV and complex psychological constructs such as self‐regulatory mechanisms and psychopathology. These findings support the idea that HRV–psychological associations are not necessarily universally consistent, but are instead influenced by a range of nuanced cultural, environmental, developmental, and methodological factors.

## Author Contributions

**Amin Sinichi:** conceptualization, data curation, formal analysis, methodology, software, validation, visualization, writing – original draft, writing – review and editing. **Georgia Eleftheriou:** conceptualization, investigation, project administration, writing – review and editing. **Mai Anh Ha Ngoc:** data curation, formal analysis, software. **Brandon A. Kohrt:** conceptualization, supervision, writing – review and editing. **Nagendra P. Luitel:** conceptualization, funding acquisition, project administration, supervision, writing – review and editing. **Rakesh Singh:** investigation, methodology, project administration, supervision, writing – review and editing. **Roxanne Jacobs:** project administration, supervision, writing – review and editing. **Katherine Sorsdahl:** project administration, writing – review and editing, supervision. **Sandra Garcia Jaramillo:** investigation, writing – review and editing. **Maria Cecilia Dedios Sanguineti:** investigation, writing – review and editing. **Crick Lund:** funding acquisition, project administration, writing – review and editing. **Mark Jordans:** funding acquisition, writing – review and editing, project administration. **Emily Garman:** data curation, writing – review and editing. **Lydia Krabbendam:** conceptualization, funding acquisition, supervision, writing – review and editing. **Martin Gevonden:** conceptualization, data curation, investigation, methodology, supervision, validation, writing – review and editing.

## Conflicts of Interest

The authors declare no conflicts of interest.

## Supporting information


**Data S1:** psyp70184‐sup‐0001‐Supinfo1.docx.

## Data Availability

The data that support the findings of this study are available from the corresponding author upon reasonable request.
