# Supplementary material for: Rethinking Resting Heart Rate Variability: No Evidence of Association With Self‐Regulation and Psychopathology in a Cross‐Sectional Study Among Adolescents in Colombia, Nepal, and South Africa
Source: Psychophysiology. 2025 Nov 14;62(11):e70184. doi: 10.1111/psyp.70184 (PMC12617397; doi:10.1111/psyp.70184)
Supplement: Supplementary file 1 — Data S1: psyp70184‐sup‐0001‐Supinfo1.docx. [file PSYP-62-e70184-s001.docx]

**Rethinking Resting Heart Rate Variability: Supplementary Material**

# Results

## Confirmatory Analysis

**Table 1** shows the full regression table for the seven models tested.

| **Outcome** | **Predictor** | **B** | **SE** | **t** | **df** | **p** | **R² (cond.)** | **ICC** |
| --- | --- | --- | --- | --- | --- | --- | --- | --- |
| **Self-regulation Domain** | | | | | | | | |
| **DERS** | Intercept | 18.77 | 2.59 | 7.25 | 1062.03 | <.001 | 0.13 | 0.02 |
|  | BMI | -0.03 | 0.08 | -0.37 | 886.09 | 0.714 |  |  |
|  | Age 14 vs 13 | 0.65 | 0.67 | 0.97 | 1091.89 | 0.331 |  |  |
|  | Age 15 vs 13 | 1.75 | 0.75 | 2.33 | 1091.15 | 0.02 |  |  |
|  | Female vs Male | 6.42 | 0.6 | 10.71 | 1090.21 | <.001 |  |  |
|  | Non‑binary vs Male | 16.82 | 4.73 | 3.55 | 594.82 | <.001 |  |  |
|  | Earlobe vs Chest HRV Method | -1.73 | 0.76 | -2.28 | 1087.26 | 0.023 |  |  |
|  | Log‑transformed RMSSD | 0.74 | 0.51 | 1.44 | 1091.49 | 0.15 |  |  |
| **False Alarm Percentage** | Intercept | 15.54 | 2.41 | 6.45 | 16.36 | <.001 | 0.07 | 0.05 |
|  | BMI | 0.01 | 0.06 | 0.18 | 661.03 | 0.854 |  |  |
|  | Age 14 vs 13 | 0.55 | 0.63 | 0.88 | 661.96 | 0.377 |  |  |
|  | Age 15 vs 13 | -0.92 | 0.68 | -1.37 | 661.96 | 0.173 |  |  |
|  | Female vs Male | -0.94 | 0.55 | -1.7 | 661.85 | 0.089 |  |  |
|  | Non‑binary vs Male | 8.33 | 3.31 | 2.51 | 661.28 | 0.012 |  |  |
|  | Earlobe vs Chest HRV Method | 0.12 | 0.91 | 0.13 | 82.79 | 0.9 |  |  |
|  | Log‑transformed RMSSD | -0.06 | 0.46 | -0.13 | 661.99 | 0.898 |  |  |
| **Immediate Reward Percentage** | Intercept | 39.35 | 7.11 | 5.53 | 159.23 | <.001 | 0.01 | 0.01 |
|  | BMI | -0.11 | 0.22 | -0.49 | 1041.25 | 0.622 |  |  |
|  | Age 14 vs 13 | 0.26 | 1.91 | 0.13 | 1038.85 | 0.893 |  |  |
|  | Age 15 vs 13 | -0.15 | 2.13 | -0.07 | 1014 | 0.944 |  |  |
|  | Female vs Male | 3.33 | 1.71 | 1.95 | 1041.61 | 0.052 |  |  |
|  | Non‑binary vs Male | -5.01 | 13.11 | -0.38 | 1041.6 | 0.702 |  |  |
|  | Earlobe vs Chest HRV Method | 0.51 | 2.09 | 0.25 | 55.68 | 0.807 |  |  |
|  | Log‑transformed RMSSD | 0.54 | 1.46 | 0.37 | 933.89 | 0.713 |  |  |
| **Psychopathology Domain** | | | | | | | | |
| **MMAPP-GAD7** | Intercept | 4.64 | 1.18 | 3.93 | 1070.81 | <.001 | 0.13 | 0.05 |
|  | BMI | -0.02 | 0.03 | -0.49 | 896.73 | 0.622 |  |  |
|  | Age 14 vs 13 | 0.13 | 0.29 | 0.46 | 1084.25 | 0.644 |  |  |
|  | Age 15 vs 13 | 0.56 | 0.32 | 1.75 | 1091.66 | 0.08 |  |  |
|  | Female vs Male | 2.23 | 0.25 | 8.74 | 1088.5 | <.001 |  |  |
|  | Non‑binary vs Male | 7.15 | 2.03 | 3.53 | 445.16 | <.001 |  |  |
|  | Earlobe vs Chest HRV Method | -0.08 | 0.33 | -0.24 | 1074.21 | 0.807 |  |  |
|  | Log‑transformed RMSSD | 0.41 | 0.22 | 1.87 | 1089.31 | 0.061 |  |  |
| **MMAPP-PHQ9** | Intercept | 6.66 | 1.4 | 4.76 | 1088.57 | <.001 | 0.11 | 0.03 |
|  | BMI | 0.01 | 0.04 | 0.2 | 1044.76 | 0.843 |  |  |
|  | Age 14 vs 13 | 0.44 | 0.36 | 1.23 | 1089.55 | 0.218 |  |  |
|  | Age 15 vs 13 | 0.47 | 0.4 | 1.16 | 1073.57 | 0.247 |  |  |
|  | Female vs Male | 2.75 | 0.32 | 8.57 | 1085.57 | <.001 |  |  |
|  | Non‑binary vs Male | 11.73 | 2.47 | 4.75 | 1084.12 | <.001 |  |  |
|  | Earlobe vs Chest HRV Method | 0.05 | 0.41 | 0.13 | 1081.83 | 0.894 |  |  |
|  | Log‑transformed RMSSD | 0.24 | 0.28 | 0.86 | 1086.97 | 0.39 |  |  |
| **MMAPP-Total** | Intercept | 14.81 | 3.47 | 4.27 | 1089.48 | <.001 | 0.13 | 0.03 |
|  | BMI | -0.03 | 0.1 | -0.27 | 1058.98 | 0.788 |  |  |
|  | Age 14 vs 13 | 1.32 | 0.89 | 1.48 | 1091.7 | 0.14 |  |  |
|  | Age 15 vs 13 | 1.76 | 1 | 1.76 | 1089.38 | 0.078 |  |  |
|  | Female vs Male | 8.13 | 0.8 | 10.19 | 1088.46 | <.001 |  |  |
|  | Non‑binary vs Male | 30.13 | 6.29 | 4.79 | 613.66 | <.001 |  |  |
|  | Earlobe vs Chest HRV Method | 0.05 | 1.02 | 0.05 | 1090.59 | 0.963 |  |  |
|  | Log‑transformed RMSSD | 0.96 | 0.68 | 1.41 | 1090.45 | 0.16 |  |  |
| **DBIS** | Intercept | 2.67 | 1.14 | 2.35 | 1048.96 | 0.019 | 0.14 | 0.12 |
|  | BMI | 0 | 0.03 | -0.15 | 540.49 | 0.877 |  |  |
|  | Age 14 vs 13 | 0.06 | 0.24 | 0.25 | 1089.36 | 0.803 |  |  |
|  | Age 15 vs 13 | 0.28 | 0.27 | 1.05 | 1090.89 | 0.294 |  |  |
|  | Female vs Male | 0.62 | 0.21 | 2.87 | 1069.06 | 0.004 |  |  |
|  | Non‑binary vs Male | 3.5 | 1.74 | 2.01 | 251.2 | 0.045 |  |  |
|  | Earlobe vs Chest HRV Method | 0 | 0.28 | 0 | 1020.6 | 0.998 |  |  |
|  | Log‑transformed RMSSD | 0.63 | 0.18 | 3.4 | 1082.03 | <.001 |  |  |

**Table 1: The full regression table** - The Outcome column shows each outcome variable related to either the self-regulation domain or the psychopathology domain. The Predictors column includes the predictors in the model, where BMI is body mass index, age is a factor (since it was a categorical variable of 13, 14, or 15), gender includes male, female, or non-binary, Earlobe vs Chest shows the method used to measure HRV (chest = Polar H10, earlobe = HeartMath), and Log transformed RMSSD is the natural log transformation of the root mean square of successive differences.

# Exploratory Analysis – Country Specific GLMs

**Table 2** shows country-specific general linear models that were fitted separately for each country. Results are shown only for the log-transformed RMSSD as the predictor due to length; however, age, BMI, and gender are controlled for in the analysis.

| **Outcome** | **Country** | **B** | **SE** | **t** | **df** | **r2** | **p (adjusted)** |
| --- | --- | --- | --- | --- | --- | --- | --- |
| **Self-regulation Domain** | | | | | | | |
| **DERS** | Colombia | -0.16 | 0.75 | -0.22 | 407.19 | 0.16 | 1 |
|  | Nepal | 0.63 | 0.86 | 0.73 | 360.98 | 0.12 | 1 |
|  | South Africa | 2.88 | 1.25 | 2.3 | 307.39 | 0.07 | 0.154 |
| **False Alarm Percentage** | South Africa | -1.43 | 0.81 | -1.76 | 270 | 0.04 | 0.56 |
|  | Colombia | 0.31 | 0.57 | 0.55 | 386 | 0.03 | 1 |
| **Immediate Reward Percentage** | Nepal | -2.59 | 2.99 | -0.87 | 361 | 0 | 1 |
|  | South Africa | 0.04 | 3.02 | 0.01 | 264 | 0 | 1 |
|  | Colombia | 2.89 | 2.01 | 1.43 | 404 | 0.03 | 1 |
| **Psychopathology Domain** | | | | | | | |
| **MMAPP-GAD7** | Colombia | -0.06 | 0.33 | -0.18 | 403.21 | 0.13 | 1 |
|  | Nepal | 0.96 | 0.38 | 2.56 | 360.98 | 0.08 | 0.066 |
|  | South Africa | 1.12 | 0.48 | 2.31 | 304.2 | 0.09 | 0.154 |
| **MMAPP-PHQ9** | Colombia | -0.22 | 0.42 | -0.54 | 407.73 | 0.17 | 1 |
|  | South Africa | 0.72 | 0.62 | 1.16 | 301.68 | 0.06 | 1 |
|  | Nepal | 0.82 | 0.47 | 1.76 | 360.98 | 0.06 | 0.48 |
| **MMAPP-Total** | Colombia | -0.61 | 1.05 | -0.58 | 407.23 | 0.19 | 1 |
|  | Nepal | 2.22 | 1.15 | 1.92 | 360.98 | 0.08 | 0.336 |
|  | South Africa | 3.58 | 1.51 | 2.36 | 306.82 | 0.1 | 0.133 |
| **DBIS** | Colombia | 0.49 | 0.28 | 1.79 | 404.49 | 0.03 | 0.518 |
|  | Nepal | 0.58 | 0.29 | 2.04 | 360.98 | 0.01 | 0.252 |
|  | South Africa | 1.19 | 0.46 | 2.59 | 298.35 | 0.04 | 0.07 |

**Table 2: Country-Specific General Linear Models Results**: The Outcome column shows each outcome variable related to either the self-regulation domain or the psychopathology domain. Each row is only the result for Log-Transformed RMSSD which is the natural log transformation of the root mean square of successive differences. Age, Gender, BMI and Device Type (ECG/PPG) are also controlled in these analyses. p (adjusted) shows the Bonferroni corrected p-values, which are the original p-values multiplied by the number of tests separately for each country (7 tests for Colombia and South Africa, and 6 tests for Nepal). Any adjusted p-value that exceeds 1 is set to 1.

# Additional Analysis

## HF-HRV

We conducted our confirmatory analyses also using the HF-HRV as the predictor. We computed the HF-HRV using the hrv-analysis Python package (Champseix et al., 2021) with Welch’s method, with the IBIs interpolated to a regular time grid at a sampling frequency of 4 Hz to compute the Power Spectral Density (PSD). The HF band was then isolated from the PSD. The power within this HF band was integrated using the trapezoidal rule to obtain the total HF power. For HF to be closely tied to RSA, the HF frequency band needs to capture the participant’s respiration rate. We lacked concurrent respiration data in the current study, as noted in the limitations section. Hence, to define the HF band, we applied two approaches: 1) We used the conventional 0.15 to 0.40 Hz band recommended by several guidelines (Laborde et al., 2017; Malik et al., 1996; Quigley et al., 2024). This is also similar to the method used in normative HRV data for adolescents used in the Sharma et al. (2015) paper; 2) To more accurately tie the HF band to respiration, we used normative values from Wallis et al. (Wallis et al., 2005) paper for the respiration band. For the age range of 13–15, respiration rates range from 13 bpm to 20 bpm for the 2.5th to 97.5th percentile, which corresponds to 0.22 to 0.33 Hz for the HF band.

The results for both HF bands are summarized below (**Table 3** and **Table 4**). For brevity, only the log-transformed HF values are shown (not the full model). The model is identical to that described in the paper. A mixed model of the pooled data with country as a random intercept, controlling for age, gender, BMI, and device type. As shown in these tables, the conclusions for both analyses (using either the 0.15–0.40 Hz or 0.22–0.33 Hz band) are identical to those reported for RMSSD in our paper; namely, no significant relationship to any of the outcome variables, except for the DBIS, which was in the unexpected direction. Please note that, given 7 tests are performed for each of these HF bands, the significance relationship for DBIS will disappear with Bonferroni correction for the HF band of 0.22-0.33 Hz (p = 0.119).

### HF-HRV (band: 0.22 – 0.33 Hz)

| **Outcome** | **B** | **SE** | **t** | **df** | **p** | **R² (Conditional)** | **ICC** |
| --- | --- | --- | --- | --- | --- | --- | --- |
| **Self-regulation Domain** | | | | | | | |
| **DERS** | 0.16 | 0.23 | 0.7 | 1091.69 | 0.482 | 0.13 | 0.03 |
| **False Alarm Percentage** | -0.11 | 0.21 | -0.51 | 661.77 | 0.611 | 0.07 | 0.05 |
| **Immediate Reward Percentage** | -0.19 | 0.65 | -0.29 | 1021.87 | 0.774 | 0.02 | 0.01 |
| **Psychopathology Domain** | | | | | | | |
| **MMAPP-GAD7** | 0.04 | 0.1 | 0.43 | 1090.97 | 0.665 | 0.13 | 0.06 |
| **MMAPP-PHQ9** | -0.06 | 0.12 | -0.53 | 1082.82 | 0.597 | 0.11 | 0.03 |
| **MMAPP-Total** | 0.02 | 0.3 | 0.07 | 1090.44 | 0.943 | 0.13 | 0.03 |
| **DBIS** | 0.19 | 0.08 | 2.39 | 1087.48 | **0.017** | 0.14 | 0.13 |

**Table 3: results for the predictor HF-HRV (band: 0.22 – 0.33 Hz)**. Age, Gender, BMI and Devices are controlled in the model.

### HF-HRV (band: 0.15 – 0.40 Hz)

| **Outcome** | **B** | **SE** | **t** | **df** | **p** | **R² (Conditional)** | **ICC** |
| --- | --- | --- | --- | --- | --- | --- | --- |
| **Self-regulation Domain** | | | | | | | |
| **DERS** | 0.32 | 0.26 | 1.26 | 1091.72 | 0.208 | 0.13 | 0.02 |
| **False Alarm Percentage** | -0.16 | 0.23 | -0.7 | 661.97 | 0.483 | 0.08 | 0.05 |
| **Immediate Reward Percentage** | -0.08 | 0.73 | -0.11 | 994.01 | 0.915 | 0.02 | 0.01 |
| **Psychopathology Domain** | | | | | | | |
| **MMAPP-GAD7** | 0.12 | 0.11 | 1.13 | 1090.01 | 0.257 | 0.13 | 0.05 |
| **MMAPP-PHQ9** | -0.02 | 0.14 | -0.12 | 1076.28 | 0.908 | 0.11 | 0.03 |
| **MMAPP-Total** | 0.19 | 0.34 | 0.54 | 1089.5 | 0.588 | 0.13 | 0.03 |
| **DBIS** | 0.26 | 0.09 | 2.84 | 1086.45 | **0.005** | 0.14 | 0.12 |

**Table 4: results for the predictor HF-HRV (band: 0.15 – 0.40 Hz).** Age, Gender, BMI and Devices are controlled in the model.

## Stability of the RMSSD Values

To evaluate whether the resting state was successfully achieved, RMSSD values were recomputed for each minute of the 5-minute recording to see if any sign of initial withdrawal and later stabilization/increase was present The results are shown in the following **Figure 1** (this is data pooled across all countries):


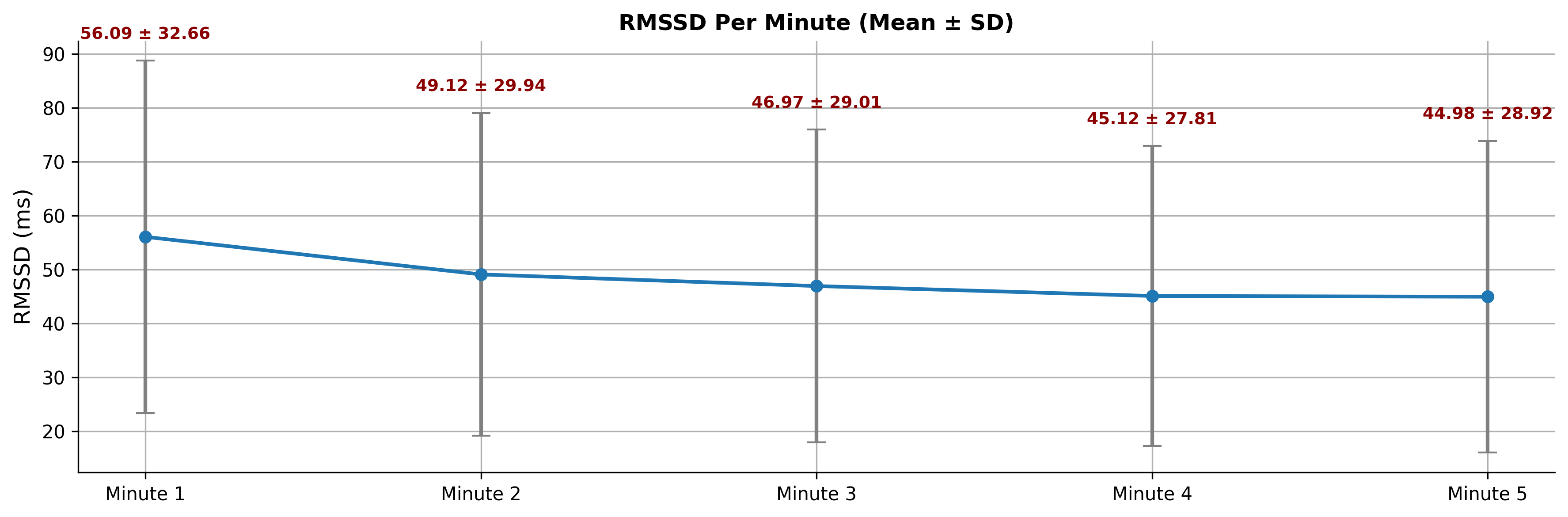


**Figure 1**: RMSSD values calculated per minute (of the entire 5-minute recording) for the entire sample.

The mean and standard deviation of RMSSD values across each minute in the 5-minute resting-state segment appear relatively similar and stable. Specifically, we do not see signs of the initial vagal withdrawal that would be evident by lower RMSSD values at the beginning and higher ones toward the end. If anything, the opposite is observed; though the magnitude of the change across the 5 minutes is small, particularly given that it is well known that even in resting-state, heart period time series are non-stationary in nature.

# References

Champseix, R., Ribiere, L., & Couedic, C. L. (2021). A Python Package for Heart Rate Variability Analysis and Signal Preprocessing. *Journal of Open Research Software*, *9*(1), Article 1. https://doi.org/10.5334/jors.305

Laborde, S., Mosley, E., & Thayer, J. F. (2017). Heart Rate Variability and Cardiac Vagal Tone in Psychophysiological Research – Recommendations for Experiment Planning, Data Analysis, and Data Reporting. *Frontiers in Psychology*, *8*. https://doi.org/10.3389/fpsyg.2017.00213

Malik, M., John Camm, A., Thomas Bigger, J., Jr., Breithardt, G., Cerutti, S., Cohen, R. J., Coumel, P., Fallen, E. L., Kennedy, H. L., Kleiger, R. E., Lombardi, F., Malliani, A., Moss, A. J., Rottman, J. N., Schmidt, G., Schwartz, P. J., Singer, D. H., & Task, F. of the E. S. of C. and the N. A. S. of P. and E. (1996). Heart rate variability: Standards of measurement, physiological interpretation, and clinical use. *Circulation*, *93*(5), 1043–1065. Scopus. https://doi.org/10.1161/01.cir.93.5.1043

Quigley, K. S., Gianaros, P. J., Norman, G. J., Jennings, J. R., Berntson, G. G., & de Geus, E. J. C. (2024). Publication guidelines for human heart rate and heart rate variability studies in psychophysiology—Part 1: Physiological underpinnings and foundations of measurement. *Psychophysiology*, *n/a*(n/a), e14604. https://doi.org/10.1111/psyp.14604

Sharma, V. K., Subramanian, S. K., Arunachalam, V., & Rajendran, R. (2015). Heart Rate Variability in Adolescents – Normative Data Stratified by Sex and Physical Activity. *Journal of Clinical and Diagnostic Research : JCDR*, *9*(10), CC08-CC13. https://doi.org/10.7860/JCDR/2015/15373.6662

Wallis, L. A., Healy, M., Undy, M. B., & Maconochie, I. (2005). *Age related reference ranges for respiration rate and heart rate from 4 to 16 years*. https://doi.org/10.1136/adc.2004.068718
